# “It's Like a Drive by Misogyny”: Sexual Violence at UK Music Festivals

**DOI:** 10.1177/10778012221120443

**Published:** 2022-08-25

**Authors:** Hannah Bows, Aviah Day, Alishya Dhir

**Affiliations:** 1 Durham Law School, Durham University, Durham, UK; 2 Department of Criminology, Birkbeck College, UK; 3 Department of Sociology, Durham University, UK

**Keywords:** sexual violence, sexual harassment, misogyny, music festivals, public spaces, live music venues

## Abstract

Despite increasing scholarly and media attention on sexual violence in public spaces, including those associated with the night-time economy and licensed venues, music festivals have been largely absent from research and policy. This paper presents the findings from the first UK study of sexual violence at music festivals, drawing on data from interviews with 13 women who have experienced some form of sexual harassment or assault at a festival. Analysis reveals that sexual violence at festivals occurs on a continuum and represents an extension of rape culture through which sexual violence is culturally condoned and normalized, enabled through a number of environmental and culture features that are unique to festivals.

It is widely recognized that sexual violence occurs in both public and private spaces; however, the majority of academic research and public/policy attention has tended to focus on sexual violence that occurs in private spaces, particularly the home (Vera-Gray & Fileborn, 2018). Despite the relative paucity of academic research on sexual harassment and violence in public spaces, anecdotal evidence, grassroots research, and industry surveys reveal that women routinely experience sexual harassment and sexual assault in public spaces, including public transport (London Assembly, 2016; SPA, 2014) university campuses (Brook, 2019) parks, and playgrounds (Budd et al., 2019). An international study of street sexual harassment in 22 countries reports that 50% of women have been fondled or groped and more than 81.5% of women have been harassed before the age of 17 (Hollaback, 2014). In the United Kingdom, 71% of women of all ages reported experiencing some form of sexual harassment in a public space, rising to 86% among 18- to 24-year-olds (UN Women UK, 2021).

Leisure spaces are frequently associated with sexual harassment and violence against women, particularly those linked to the night-time economy (NTE), situated within the “sexualized city” (Hubbard & Colosi, 2012). The existing work has illuminated the role that commercialized and heteronormative spaces play in contributing to developing “cultural atmospheres” (Kavanaugh, 2013, p. 242) where unwanted sexual attention becomes accepted as a normal part of being in these spaces (see Brooks, 2011; Fileborn, 2016; Gunby et al., 2020; Quigg et al., 2020; Sheard, 2011). In the United Kingdom, a Drinkaware study in 2015 reported 54% of women and 15% of men aged 18–24 experienced sexual harassment on a night out. Mellgren et al. (2018) found that sexual harassment of Swedish women occurred frequently at clubs and restaurants. More recently, Miranda and van Nes (2020) report that nearly all sexual violence cases occurring in public spaces at night are close to, or offer, nightlife activities. Moreover, emerging research in the context of live music venues has documented the routine everyday occurrence of harassment and sexual violence experienced by women in these environments (Hill et al., 2020). Thus, as Pain (1991) pointed out, while research may indicate the majority of (interpersonal) violence takes place in the private sphere (usually the victim's home), this does not mean the public sphere is a safe place for women.

Culturally and economically, music festivals are important British leisure events, at the “heart” of the British summer time (McKay & Webster, 2016). The turn of the century saw a massive expansion of the outdoor music festival scene into a large-scale commercial and corporate sector (Morey et al., 2014). Mintel (2018) estimates that there were 918 UK festivals in 2018, more than double that of a decade earlier, while CGA (2019) estimates more than 7.1 million people attended a festival in 2018. Many contemporary music festivals promote themselves by promising freedom, excitement, and hedonistic pleasure, thus encouraging consumers to purchase the experience of 1960’s hippie counterculture. “Freedom” is commodified as central to the marketing of many music festivals, which now form a highly commercialized sector of the UK leisure industry, subject to various regulatory restrictions (Griffin et al., 2018, p. 1). However, as festivals vary significantly in terms of size and audience make up, such as by age and the music genres or cultures being sought, it is important not to homogenize “the music festival.”

Demographically, the festival landscape has also shifted in recent years. Although music festivals were traditionally dominated by men, 60% of UK festival visitors in 2016 were female, an increase from 37% in 2015 (Statista, 2016). Elsewhere, women make up almost half of festival audiences. In Australia, 44.3% of festival attendees in 2018 were female (Hughes et al., 2019) and across European festivals in 2016 just over 45% of attendees were female (Statista.com, 2021).

Despite women occupying increasing space at festivals, the emerging research indicates they are not free to experience these events in the same ways as men (Aborisade, 2021; Baillie et al., 2021; Fileborn et al., 2020; Wadds et al., 2022). The two studies outside of the UK examining sexual violence at festivals through interviews with survivors (Aborisade, 2021 in Nigeria and Fileborn et al., 2020 in Australia) report on the normalcy of sexual violence in festival spaces, low reporting to festival staff or other formal agencies, and a range of negative consequences as a result of their experiences. These findings are supported by data in the United Kingdom and the United States. In the United Kingdom, a YouGov survey in 2018 reported that sexual harassment is prevalent at UK music festivals, revealing 43% of female festival goers under the age of 40 experienced unwanted sexual behavior at a music festival but only 2% reported to the police (although 7% did report it to festival staff). Our research has revealed similar findings, with 34% of women and 6% of men respondents experiencing sexual harassment at a festival in the previous five years, and 9% of women and 1% of men experiencing sexual assault (Bows et al., 2022). Interestingly, these reported levels are significantly lower than the figures reported in a survey of festival attendees in the USA, which revealed more than 90% of female respondents said they had been sexually harassed at a music festival or music gig/venue (OMMB, 2017).

Although there has been very little academic research on sexual violence at festivals, the issue has received high-profile media coverage over the last few years (Davies, 2017; Owen, 2021), leading to grassroots campaigns and activism (e.g., Girls Against and Safe Gigs for Women), in some cases, extraordinary responses from festivals. For example, in 2016 and 2017, a spate of rapes and sexual assaults of women at Bravalla, a Swedish festival, were reported, which led to the cancelation of the event in 2018 until men could “learn to behave themselves” (O’Connor, 2017). Similarly, Clear Lines Festival was set up in 2015—founded and crowd funded by volunteers—with the specific goal of addressing sexual assault and harassment through the arts and discussions. In the UK, the Association of Independent Festival led a campaign where music festival websites went dark for 24 h to raise awareness of sexual violence and call for positive action across the industry (Moore, 2017).

While the survey findings and media reports give some indication of the extent of sexual harassment and assault at festivals, there are major gaps in knowledge about the prevalence, nature, experiences, and responses to sexual violence. This article presents data from the first UK study to explore women's experiences of sexual violence at UK music festivals, contributing to the small but growing body of literature in this area (Aborisade, 2021; Baillie et al., 2021; Bows et al., 2020, 2022; Fileborn et al., 2020) and to the broader literature on gendered experiences of leisure spaces (Aitchison, 1999; Green & Singleton, 2006; Gunby et al., 2020; Hill et al., 2020; Stevenson, 2017).

## The Study

Drawing on data from a larger study exploring the extent, nature, scope, and responses to sexual violence at UK music festivals (Bows et al., 2020, 2022), this article focuses on qualitative interviews undertaken with 13 victim-survivors of unwanted sexual attention at festivals. As this part of the study focused on subjective, situated experiences of sexual violence, an inductive, qualitative approach was adopted. The study is situated within a feminist framework, which foregrounds the role of patriarchy in providing a heteronormative framework for men's violence against women, including sexual harassment and assault (Gavey, 1992). Ethical approval for the study was granted by Durham Law School Ethics Committee.

Participants were primarily recruited through earlier methods of data collection in the larger study, namely an online survey which collected data on the nature/scope of sexual violence at festivals (Bows et al., 2022). At the end of the survey, participants were asked if they would be willing to take part in interviews to discuss their experiences of sexual violence at festivals and all of those who provided contact details and permission to contact them were subsequently emailed and invited to take part in an interview. Social media, particularly Twitter and Facebook, were also used to promote the study and recruit participants. The sample was therefore purposive and self-selecting.

Although the study was open to participants of all genders, only female survivors gave permission to be contacted or responded to the social media calls for participants. In total, 13 women who had experienced some form of sexual harassment or assault at festivals took part in interviews. The age range of participants was between 18 and 40. While the sample is not representative of all female festival-goers, it captured those with lived experience—from whom detailed insight can be developed (Hesse-Biber, 2011). To protect the identity of participants, pseudonyms are used for all quotes.

All participants were provided with an information sheet and consent form prior to the interview. Signed consent forms were obtained from all participants. Although participants were offered the opportunity to conduct interviews face-to-face (with travel expenses covered), all opted to have the interview over the telephone. Interviews lasted between 30 and 80 min. A semi-structured interview schedule was used to ask broad questions about experiences of sexual violence and responses (their own as well as formal and informal) at festivals. All participants were given a high-street voucher as a thank you for taking part in the research.

Telephone interviews were typed at the point of the interview producing immediate transcripts. Transcripts were analyzed using a critical feminist framework; the subjective experiences of women were central to the analysis, which was concerned with developing rich descriptions of experiences of sexual harassment and violence, the impacts and reporting decisions, and responses (by participants and others). Coding began by reading the transcripts line by line and attaching descriptive labels to each phrase, sentence, and later to larger sections of text to identify and interpret the experiences and perspectives of survivors (Ritchie & Spencer, 2002). Coding was initially done by the lead researcher and subsequently checked and confirmed by two other members of the research team. Themes were then drawn out of the transcripts based on these codes.

This work is foundational, as there are no previous studies in the United Kingdom examining sexual harassment and violence at music festivals, and only two other published academic studies outside of the United Kingdom (Aborisade, 2021; Baillie et al., 2021; Fileborn et al., 2020; Wadds et al., 2022). Through our analysis and presentation of our findings in this paper, we hope to kickstart a fuller discussion of the emerging issues in order to establish a body of research examining, empirically and conceptually, sexual violence at festivals and live-music spaces.

## Findings

This section presents the analysis of women's accounts of sexual harassment and violence at UK music festivals. Most of the participant's reflections refer to experiences at large commercialized festivals—which make up a significant proportion of the festival landscape in the United Kingdom—although one example did occur at a small, independent festival.

Four themes were identified in women's narratives of their experiences and responses. These are (a) a continuum of sexual violence at festivals, (b) cultural atmospheres, (c) rape culture, and (d) tactics to reduce sexual violence at festivals.

### A Continuum of Sexual Violence at Festivals

Consistent with the literature on sexual violence in other live music venues (Hill et al., 2020), festivals (Fileborn et al., 2020), and nightlife settings (Huber & Herold, 2006), acts of groping at festivals were particularly common among our participants. All the participants in our study described instances of being brushed up against, rubbed, or “groped while you are going through the crowd or grinding on you from behind” (Kim). Consistent with the wider literature on sexual violence in public spaces, particularly live music and nightlife venues (Fileborn, 2016; Hill et al., 2020), all of the harassment and assaults described by participants were perpetrated by either (male) strangers or acquaintances.

Several participants described incidences of sexual violence that would meet the definitional threshold for some of the most legally serious offences, including penetrative assault:

I was watching the mainstage and very into it, as we often are in our own worlds doing your thing, just living. He just appeared out of nowhere and I was wearing a skirt and he whacked his hand up my skirt and his fingers up inside me and picked me up off the floor by my genitals. (Orla)

Another participant shared a similar experience:

I was in one of the venues stood with a friend, stood chatting, with a bunch of guys. I just felt a hand up my skirt who full-on grabbed my crotch. (Becca)

Some of the incidents described by participants in our study met legal definitions/categories of sexual harassment and assault, but others were less easily identified. Speaking about a security guard who was moving the participant and her friends away from the stage where they had been dancing, Annabel described an incident of verbal sexual aggression:

And then he whispered in my ear behind me and this is not a quote but it was like “don’t make me spank you, you naughty girl, you want me to spank you don’t you, don’t make me come over there and spank you, you naughty…”

Verbal sexual violence and harassment were also described by Addie:

The one that sticks out the most it was a festival I first went to. I was 15, on boundary of 15 and 16. We were walking to our tent. And we all looked underage and a man in his mid to late 20s and came up to my friend and said “I bet you’d like to feel my beard tickling between your legs.”

Wider research on sexual harassment and violence in nightlife settings has reported the common and frequent occurrence of these routine intrusions. For example, Huber and Herold (2006) report that 82.5% of women have experienced buttock touching in bars, while Graham et al. (2014) report 50% of women experienced unwanted or persistent sexual aggression on a single night out. These gender microaggressions (Sue, 2010) can be understood as forms of sexist violence, which function as a potential “gateway mechanism,” with research documenting gender microaggressions, sexual harassment and sexual assault exist “along a continuum from chronic, low-severity to infrequent, ‘high severity’ offenses” (Gartner & Sterzing, 2016, p. 491). Julia provided an example of gender microaggressions through persistent objectification by a (male) colleague, while she was working at festival, who would routinely comment on her body, in particular the size of her breasts, which escalated to other forms of sexual harassment:

The thing I go to when I speak about this kind of thing is a dude I worked with in the music industry who made comments about the size of my tits when we were hanging out at a music festival. So that was sexual harassment. And then it was an ongoing thing of him continually asking me to sleep with him and me saying no and him not listening to the answer no. That was on going over a period of months and that was quite a big one.

Several other women gave accounts of casual sexism as “gateways” into further harassment and in some cases assault. Addie recounted an experience where a group of men had followed her and her friends at a festival, initially trying to start conversation and when ignored, made sexist comments and sexually harassed the women. Sarah described a man who pushed into her in the crowd several times and initially claimed it was an accident but then went on to make sexual references and grope her.

This type of chronic and persistent harassment is well documented in research exploring women's experiences of sexual aggression (see, e.g., Hlavka, 2014). These experiences may be further understood through the prism of objectification theory, introduced by Fredrickson and Roberts (1997) to describe and explain a culture of sexual objectification in which women are treated as (sexual) objects or things, rather than as people (Davidson & Gervais, 2015). Sexual objectification experiences occur on the continuum of sexual violence (Kelly, 1988), incorporating the everyday microaggressions and “sexist violence” (Hill et al., 2020) described by our participants such as saying “suggestive stuff” (Addie) or making comments about Julia's body and breasts through to the groping, assaults, and attempted rapes described by our other participants.

### Cultural Atmospheres

The participants in our study identified several features of festivals that they felt contributed to cultural atmospheres of sexual violence. Although sometimes described separately by participants, these features intersected and overlapped to create particular environments and atmospheres at festivals conducive to harassment, sexism, and sexual violence.

For example, reflecting on the physical size of some of the larger festivals in the United Kingdom, where tens of thousands of people come together for events that span multiple days, Becca explained:

I think on nights out people are encouraged to be a bit silly but it is for a small amount of time, easy to contain in a small number/bar with limited capacity. When you get to festival it's on much wider scale, and it is encouraged. (Becca)

Here, Becca describes the importance of the physicality of the festival space as well as the cultural encouragement of behaviors—“being a bit silly.” This was articulated by several participants, who identified the combination of large numbers of people, dense crowds, intoxication, and the marketing of festivals as hedonistic and escapist as producing cultural atmospheres within which sexual violence materializes:

People use the crowded spaces as a way of getting away with a lot of stuff, like a lot of people I know have had drinks spiked with drugs and people just do it for a laugh or to try and get with someone. That atmosphere – laid back, crowded, trying to meet new people, it gives the perpetrator a viable reason in their mind to do what they are doing. (Sarah)

The man can be anywhere and if you’ve had the courage to speak to staff, it could have happened to like three other people in that time. It's like a drive by misogyny. It is very drive-by … but I guess I’ve never thought about the festival space as somewhere where you can do something and then just escape and disappear. (Julia)

The physical layout and design of festivals thus provide opportunities to perpetrate sexual violence undetected. Previous research on violence at festivals has similarly noted that the sense of anonymity among large crowds may obfuscate groping as “accidental” and even “unavoidable” aspects of the festival experience (Fileborn et al., 2020). Anonymity is further extended by the fact that many festivals occur into the evening, providing a cloak of darkness for perpetrators in crowds which are often characterized as chaotic spaces (Fileborn et al., 2020), which can legitimize criminal and abusive behavior including groping. Moreover, festival spaces are transient and can change in their layout from year to year, or even day to day. Spatially, this distinguishes festivals from other live music events and the nighttime economy more broadly, which are located within permanent structures. As Carrie put it, “it's different at festivals [compared with nightclubs] because you don’t have a designated safe space you can get to.”

The spatial design and cultural features of the festivals described by participants are echoed in research examining women's experiences of other leisure spaces, particularly the night time economy (Wadds et al., 2022). In particular, recent research on masculinities and lad culture has illuminated the importance of cultural and spatial contexts of sexism, harassment, and sexual violence (Kavanaugh, 2013). Lad culture is a term to define a “set of practices, behaviors and activities often associated with University-aged men and characterized by a homosociality that endorses, in different degrees, misogyny, sexism, homophobia and, in its most extreme cases, sexual violence” (Diaz-Fernandez & Evans, 2020, p. 744). Some of the core traits, or behaviors, of lad culture including excessive alcohol or drug consumption, escapism, and hedonism (Gill, 2003) are also components of festival culture. Indeed, scholars have recently argued that misogyny and sexual violence at festivals must be understood through an appreciation of wider cultural practices at festivals. Wadds et al. (2022) have argued that the sense of escapism and liminality central to the festival experience and the use of drugs and alcohol, which is part of the carnivalesque ritual associated with festival attendance, contribute to a culture of sexual violence. Our participants also felt that many of the characteristics of festivals that were attractive to attendees were also part of the “problem” of sexual violence. Orla described festivals as offering a “condensed space where you can experience traveler culture and different ways of living inside or outside society” and normal rules do not apply. Addie agreed:

I think it’s a lot to do with the culture when you go a festival. Some people have the attitude that it’s a lawless land. Which goes in hand with drug use which isn’t monitored much [at festivals] and it’s a happy-go-lucky do whatever you want attitude.

Kim also described consumption and hedonism as key features of festivals that also contribute to a culture where sexual violence is expected:

And people do get drunk and do take drugs and they do lose their inhibitions and it might not be like that in everyday life that they will be that person. But when you’re combining the two and you’re partying hard and inhibitions go … it’s not acceptable but it happens. (Kim)

For some participants, misogyny and sexual harassment were considered *inherent* to the culture of festivals and the wider music industry, which is run by men, for men, and thus endorses and promotes lad culture. Speaking about her experiences across multiple festivals, Julia reflected:

There isn’t a particular music festival where it’s happened more. Not really. I think it’s a thing that’s inherent within festivals and party culture. When you’ve got a massive party that is run by a music industry bro, that’s the culture, that’s the people in the industry running it. I feel like it’s specific to any one festival, I think it’s across the board.

### Rape Culture

For participants in our study, there was a shared expectation of sexual violence at music festivals. This was informed by their broader understandings and experiences of gender-based violence which were framed by the broader heteronormative, patriarchal culture in which sexual aggression is routine and common place. In the patriarchal culture, men's violence against women is viewed as customary and normal, and thus so is women's endurance of it (Stanko, 1985).

This was articulated by participants, who referred to the normalization of sexual violence in their life—something that women expected and had been socialized to see as normal, just something that happens (Kelly & Radford, 1990)—which shaped their understandings and narratives of sexual violence and harassment at festivals. The women we spoke to gave accounts of their experiences of misogyny and sexist violence both before, and during, music festivals. Orla described this as a part of her “gendered culturing”:

Partly I think because of how we are gendered, we’re cultured through our gender so sexual violence, like some people have a better compass but we’re kind of taught to expect that. I had someone put their hands down my trousers at work when I was a waitress and my boss told me “that’s just part of the job.” So partly I think that’s just what we’re told to expect and deal with. But, also, I don’t think we’re taught that that’s assault.

Other women, like Becca, described sexual harassment and sexually aggressive behaviors as routine and customary, “that's what life's like for a teenage girl, suck it up.” Participants in our study described becoming numb to sexual harassment and violence, something that “as a woman you learn to ignore” and “just fade them out eventually” (Lily). As Annabel explained, sexual harassment and aggression become part of women's gendered identity, “it just slides and it slides and you take it on and it becomes part of you and people being mistreated and people talking to you like however the fuck they want. Because you’re a woman.”

Women's subjective experiences of sexual aggression at festivals were thus bound up with their previous, everyday experiences of men's sexual objectification and harassment. In other words, participants described sexual violence at festivals as an extension of “rape culture” that permeates every aspect of their lives. As Annabel reflected:

So many times you’re in a nightclub or a festival, anywhere … people they say shit to you, they’re in authority like police officers or security or even just like men who think they own shit, they violate you in one way or another by a look, a phrase, cat calling you and you feel completely helpless, there’s nothing you can do.

Rape culture was first introduced by Brownmiller (1975) and expanded by Buchwald et al. (1993, p. vii) who define it as the

complex of beliefs that encourages male sexual aggression and supports violence against women. It is a society where violence is seen as sexy and sexuality as violent. In a rape culture women perceive a continuum of threatened violence that ranges from sexual remarks to sexual touching to rape itself. A rape culture condones physical and emotional terrorism against women as the norm.

In the context of music festivals, rape culture is identified through the continuum of misogyny, sexual harassment, and sexual violence experienced by women, which is subjectively, yet collectively, understood through the gendered prism of women’s wider experiences of patriarchal structures within which sexual harassment and violence are normalized and routine. Women had come to expect sexual violence at festivals, as they did in other spaces, as part and parcel of being a woman.

### Women Adopt a Range of Tactics to Reduce the Risk of Sexual Violence at Festivals

The consequences and impacts of experiencing these acts of misogyny, harassment, and sexual violence at festivals varied. Several participants told us they felt shocked and upset. Becca said she thought she would react differently if it happened now “but at the time I was so shocked.” Sarah described similar findings:

At the time I was a bit, kind of, devastated, upset, those initial emotional experiences. Like, nothing is being done about this. I don’t feel comfortable being here and wanted to go home.

Hill et al. (2020) argue that sexist and sexual violence against women in live music venues serve to control of women in a particular space, limiting their access to participate freely and fully in the experience—hindering their ability to lose themselves in the music, which is one of the joys of experiencing live music. This is echoed by the accounts of women in our study. Annabel and Sarah described not being able to enjoy the festival after the incident:

I remember feeling violated. I was really shocked, and I was just like speechless. I couldn’t just get back into dancing and having a good time. I just kept feeling like it wasn’t right at all, but then I remember feeling really conflicted with all the other emotions that came through with it. (Annabel)

I wasn’t crying, but I was definitely upset and it had ruined the experience. (Sarah)

As a result of the routine experience of sexual violence that our participants had come to expect, several women in our study described ignoring or brushing off the harassment or assault they experienced at festivals:

We just brushed it off and carried on, I think most girls get used to hearing creepy comments from men so we become immune to it after a while. (Lily)

This was similar for Becca:

I just thought at the time I can’t be bothered dealing with it. My mentality is different, I think I thought “shit happens.”

As others have noted about sexual violence in universities and leisure spaces, the ubiquity of sexual harassment is one of the reasons such incidents are often not reported, or responded to formally by women (Jackson and Sundaram, 2020). However, this does not mean women “do nothing.” Indeed, our participants told us about the extensive efforts they went to in order to avoid or reduce the risk of sexual aggression at festivals. Women told us they adopted a number of strategies to protect themselves against expected sexual harassment and aggression. This included avoiding certain spaces, ensuring they/their friends were never on their own, or reducing their alcohol consumption. These risk assessment and management strategies are part of what Vera-Gray & Kelly (2020) refer to as “safety work,” which women constantly undertake to minimize the risk of men's violence in public spaces. For one participant, the incident resulted in them avoiding festivals altogether:

I’ve had to stop attending music festivals this year because I was having really bad anxiety attacks whilst there and ended up not enjoying myself. I think there's also a slight fear, for me anyway, of something bad happening at festivals, and I can’t pinpoint exactly what it is. (Lily)

Other participants similarly described a lingering fear:

There's always a fear, even related to that, incidents of crime unrelated to sexual violence, when a friend who has had their tent rummaged through it brings on a fear of sexual violence especially for the females in the group. (Carrie)

Consistent with Sheard (2011), Gunby et al. (2017), and Sundaram and Jackson (2018), women in our study found ways of avoiding or mitigating the risk of sexual harassment or violence by modifying or restricting their practices and movements. Some women told us they had responded directly to the perpetrator, calling out their behavior or challenging them:

I restrained him, screamed at the top of my lungs for [friend's name]. I had his hand up his back restrained, and I pushed him out the door which was zipped up. [Friend's name] came and, she ripped the door open because she couldn’t get the zip open quick enough, dragged him out by his hair, screaming at him to go away. (Kim)

However, these actions were not always successful; despite directly challenging the perpetrator, the behavior continued or escalated in several incidents. For example, the incident above where a man came into the participant's tent and she subsequently restrained him and screamed until a friend came to assist did not result in the immediate desistance of the perpetrator's behavior. Several other participants described violence escalating from initial unwanted comments or attention to attempted, or successful, assaults. This is consistent with the wider literature of sexual violence in public places, which has identified that harassment is often a precursor to more serious violence (Adur & Jha, 2018). This highlights the risks associated with resisting and challenging misogyny and violence and underscores the importance of collective responsibility for preventing and responding to sexual violence so that it is not left to individual women to prevent men from harassing and abusing them (see Baillie et al., 2021).

Some participants felt unable to challenge such behavior, for fear of the violence escalating. One woman said she felt any attempts to challenge men's unwanted advances would likely lead to the labelling of being a “raging feminist”:

I have seen so much sexual harassment working on bars at live events and it's just considered the norm, and no one ever does anything about it because you’re seen as being “too raging feminist” or “too sensitive.” (Lily)

Tomlinson (2010, p. 1) notes the trope of “the angry feminist” is ubiquitous in contemporary society and is “designed to delegitimize feminist argument even before the argument begins” serving as “discursive technologies of power that accounts for, swallows up, deflects, deflates and redirects social criticism” (p. 31). It is a powerful tool for silencing those who speak about sexual violence, even before they have spoken, and is an important feature of rape culture. As Hlavka (2014, p. 340) notes, “multiple discursive strategies and ideologies have operated to undermine or dismiss survivor's speech” and the silencing of women is part of rape culture, which normalizes and condones violence against women. Phipps and Young (2015) describe this as postfeminist irony—an inbuilt defense strategically deployed as a shutdown critique where sexism is trivialized so that those who challenge it are positioned as kill-joys and the label “feminist” is a denunciation.

## Discussion

This article presents the findings from the first UK study examining women's experiences of sexual violence at music festivals, contributing to the small pool of research examining this issue internationally (Fileborn et al., 2020; Wadds et al., 2022). Consistent with the research on sexual violence in other nightlife settings, women in this study described a range of behaviors experienced at festivals, including verbal and physical sexual harassment and cat calling, groping, sexual assault, and rape (Brooks, 2011; Gunby et al., 2017, 2020; Hill et al., 2020).

All of the incidents described by participants were perpetrated by men. Many of these behaviors are particularly common in other nightlife settings, such as bar, clubs, and gig venues where catcalling, harassment, and groping are routine, and in many cases expected (Brooks, 2011; Fileborn, 2016; Gunby et al., 2017, 2020; Hill et al., 2020). Similarly, participants described the gendered normalcy of harassment and groping, both at festivals but also in other spaces, where women have learnt to expect these intrusions. Thus, sexual violence at festivals is not distinct from the sexual violence women experience at other times in other spaces; rather it is on the continuum of sexual violence that women experience across multiple times, spaces, and life stages. Moreover, women's understandings and narratives about sexual violence at festivals were informed by their broader, gendered, situated experiences of public (and private) spaces. As one participant put it, she had been through a “gendered culturing” to expect sexual harassment and violence, and our other participants described sexual violence as every day, routine, experiences. These are part and parcel of being a woman, occupying (male) spaces.

While misogyny and sexual violence are not specific to festivals, there are cultural and physical features of the festival environment, which enable these actions. Several defining characteristics of modern commercialized festivals in the UK—hedonism, liminality, boundless spaces where problematic alcohol and drug consumption is normalized and excess is encouraged—are also observed in broader problematic “lad cultures,” which are arguably constructed and reproduced in festival environments and spaces. Our participants also identified several spatial features specific to the festival environment—the design of festivals provides opportunities to harass and assault women with relative anonymity and in turn making reporting difficult. The combination of these cultural and spatial features results in festivals producing a singular space, which facilitates sexual violence. Gisbert and Rius-Ulldemolins (2019) argue that the “rhetoric that labels festival seasons as exceptional circumstances in which conventions are abandoned and freely enjoyed legitimizes patriarchal behavior towards female bodies” (p. 4).

Similar to how Valentine (1990) established that areas such as multi-story car parks, alleys, and open spaces leave women feeling vulnerable, the festival environment too has identifiable features that participants associated with sexual harassment. Crowds offered perpetrators the ideal opportunity to sexually harass and assault women with relative anonymity and therefore impunity. One of our participants likened this to “drive by misogyny,” as perpetrators are able to harass and assault and then quickly escape undetected. In this sense, we can see that festival space replicates the conditions of the urban space, with Lewis (2018) identifying how perpetrators exploit the transitory, anonymous nature of urban public transport to “slip way,” leaving survivors with the impression that the harasser is untraceable.

Similarly, the cultural features and practices which attract many women to festivals were also perceived to increase the risk of sexual harassment and assault—creating what Kavanaugh (2013, p. 242) describes as “cultural atmospheres.” Women described festivals as offering alternative spaces, free of the restrictions and mundanity of everyday life, where “anything goes.” Festivals are broadly considered carnivalesque events, where people, particularly young people, seek out the opportunity to transgress the restrictions of normal life, creating (sub)cultures of liminality within which different norms and identities are carved out (Dilkes-Frayne, 2016). In these settings, alcohol and drug intoxication are common and encouraged, close contact with others is and as one of our participants described, the festival space feels like a lawless land where normal rules do not apply.

We are reminded here of the work of Diaz-Fernandez and Evans (2020) who explored gendered accounts of the NTE and described how such spaces create “sticky atmospheres,” which affect how women and gender-minorities experience and negotiate these spaces. We concur with Diaz-Fernandez (2020) that affect is plugged into the contextual and that spaces—in this case the modern commercialized festival spaces—shape masculine and “laddish” performances and women's experiences. However, it is important to avoid associating deviant or counter-cultural practices with sexual violence. Festival spaces vary greatly with some women reporting being attracted to certain drug-taking and music scenes because a deemphasize on machismo is freeing (McRobbie, 2000) while serious sexual violence has been reported at festivals marketed as “family friendly” (BBC, 2017).

These, in turn, connect to and are located within a wider patriarchal society that provides the scaffolding for a “rape culture” (Brownmiller, 1975) within which “women perceive a continuum of threatened violence that ranges from sexual remarks to sexual touching to rape itself” and both physical and emotional terrorism against women is the norm (Buchwald et al., 1993, p. vii). The normalization of sexual harassment and violence of women, both within festival spaces and across wider society, and the routine minimization of women's experiences and low levels of reporting provide the cultural scaffolding (Gavey, 2005) for sexual violence to be perpetrated and simultaneously ignored or denied.

Consistent with the research exploring how women navigate public spaces generally, and licensed venues specifically (Armstrong et al., 2011; Brooks, 2011; Diaz-Fernandez & Evans, 2020), our research found that women undertake a significant amount of “safety work” (Vera-Gray & Kelly, 2020) to reduce the risk of sexual violence at festivals. This included reducing their alcohol consumption, avoiding certain spaces and, in some cases, avoiding festivals altogether. These strategies and tactics are routinely adopted by women to manage their safety and shape the affective atmospheres in order to control or evade lad culture (Diaz-Fernandez & Evans, 2020). It is common for women to restrict and adjust their behavior and movement through public spaces, both physically and symbolically, in order to manage the fear of encountering sexual violence (Pain, 1991; Stanko, 1993; Vera-Gray, 2018). Much of this “safety work” involves women being hypervigilant and making subtle behavior adaptations involving the way certain places and times are negotiated (Pain, 1995; Valentine, 1989; Vera-Gray, 2018).

Some other women described a more direct resistance to the culture of sexual harassment and violence at festivals, by challenging unwanted behavior. These findings chime with research on gendered experiences of the NTE, which has documented women's resistance to unwanted sexual attention in these spaces (Graham et al., 2017; Gunby et al., 2020). Although it is widely acknowledged that public spaces, and in particular nightlife settings and live music venues (Hill et al., 2020), continue to be “masculine republics” (Harrison, 1971, p. 47) where women's access, behavior, and opportunities are controlled by the men who created and dominate them, post-structural feminists have suggested these sites provide opportunities for resistance, providing women with a platform to contest and challenge cultural constraints and discourses (Brooks, 2011). This type of direct resistance has been described as a form of “feisty femininity” (Gunby et al., 2020). Thus, women are not simply passive subjects of (male) violence at festivals, but rather engage in safety work to reduce and manage their risk as well as directly challenging sexism and misogyny. However, resisting sexual violence itself is associated with further risks—it is well established that refusing men's unwanted advances can lead to further abuse and escalations in violence (Stratmoen et al., 2018; Wesselmann et al., 2010) and women often adapt their behavior accordingly, finding “polite” ways to reject unwanted advances (Stratmoen et al., 2020). Several women in this study described men continuing to harass them after being rejected, and in some cases, this led to an escalation in the violence and abuse.

Some women described more general discomfort, a lingering fear of violence or harassment at festivals, even when they employed various tactics and strategies to reduce the risk. This lingering fear and anxiety and adaptations of behavior connect with the broader literature, which has shown space, particularly public spaces, are gendered in their construction and subjective experience. The constant threat of sexual violence “works to change the demographic constitution of the space, making women feel unwelcome” (Hill et al., 2020, p. 375).

Festival organizers sell “countercultural carnivalesque” (Anderton, 2008) while obscuring or ignoring sexual violence by minimizing women's complaints as “too raging feminist” as one respondent called it—suggesting that to confront the issue would allow “feminist killjoys” (Ahmed, 2012) to unmask the façade. It can be argued that the overtly masculine framework of which a majority of mainstream festivals take place permits this behavior, encouraging and (re)producing laddism and rape culture more broadly. We concur with Wadds et al. (2022, p. 2) who argue that “highly gendered, destructive and violent aspects that have always featured at carnivalesque events, including the sexual violence and harassment that is born from the unbridled escapism and rule-breaking that the carnival facilitates and, indeed, often actively promotes.” Ultimately, despite the increased presence of women at music festivals, they continue to be patriarchal spaces and women's experiences are still structured by gendered ideals and stereotypes of normative femininity and (hetero)sexuality and vulnerabilities to sexual harassment and assault. They are not free to enjoy festivals in the same way as men.

## Implications for Policy and Practice

While participants described misogyny and sexual harassment as ubiquitous at neoliberal, commercial festivals and beyond, the unique spatial and culture features of festivals open opportunities to change the design of festivals and in turn “unstick” the atmospheres, or make them *stick differently* (Diaz-Fernandez & Evans, 2020, p. 760, original emphasis). One of the unusual features of festivals compared with other nightlife and live music settings is that the venue is not fixed; it is changeable and adaptable, providing opportunities to (re)design and reimagine the spatial layout of festivals.

However, such spatial changes must go beyond the simple modification of the physical space to incorporate social and cultural dynamics and relations. There have been limited studies on the possibilities and effects of situational crime prevention for reducing sexual violence, although some recent work has suggested that the presence of guardians—that is, third-pa–ties—has a positive influence on the outcomes in sexual offences (Beauregard & Leclerc, 2007; Cook et al., 2019) and may offer opportunities for prevention at festivals (Baillie et al., 2021). Yet, others have warned that the presence of other people is not sufficient in and of itself—rather, the setting and context interact with the presence of others to (potentially) increase, or reduce, the incidence of sexual violence (Fileborn, 2016). Moreover, the presence of security officers patrolling public locations, nearby residents/businesses, and visibility may be effective in reducing the incidence of sexual violence in public spaces (Gekoski et al., 2015), but these studies have focused on fixed spaces, and our study as well as Fileborn et al. (2020) found that security staff (and other festival staff) are sometimes the perpetrators of sexual violence. Furthermore, BeebeeJaun (2009) points out that most situational crime prevention techniques, such as CCTV, are not suitable to monitor verbal and less explicit forms of sexual violence, which constitute the majority of experiences women are subjected to in public spaces and formed a significant proportion of the incidents participants told us they had experienced at festivals.

Further research that examines the potential for sexual violence prevention through the mediation and manipulation of physical space is needed, but this must take into account broader social and structural issues which contribute to cultures of masculinity and misogyny at festivals. Over the last few years, there has been an increase in festivals recognizing sexual violence and committing to developing policies and practices to improve women's safety. For example, the Association of Independent Festivals (AIF) developed a charter of best practice which takes a zero-tolerance approach to sexual violence at festivals and commits to raising awareness and providing training to staff and volunteers to enable them to report concerns and support victims who make disclosures. Several organizations and campaign groups, for example White Ribbon UK, have also been involved in campaigns to raise awareness of sexual violence at festivals and call for festival companies to take action. However, most UK festivals still do not acknowledge the issue of sexual violence and few have yet to implement policies or introduce initiatives to reduce the incidence. It is crucial that this work is undertaken and should involve specialist support groups, such as Safe Gigs for Women, to develop and deliver training as well as dedicated support services on-site at festivals to respond to victim disclosures. One way of achieving this in the UK would be for local councils and relevant agencies who grant licenses for festivals in public spaces which require festival organizers to comply with extensive conditions concerning safety, security, and welfare at festivals, to require festivals to specifically include policies and associated practices for sexual violence prevention and support provision for victims.

There are a number of limitations with our study. The sample size was relatively small and consisted of predominantly white, heterosexual women. Research which incorporates, or specifically focuses on, the experiences of women, and men, from diverse communities is thus required to expand our understandings of sexual violence at music festivals. Furthermore, our participants described spatial and cultural issues with festivals, which are observed primarily at large, commercialized festivals in the UK. Research that examines experiences at a wider range of festivals will be useful in identifying whether these spatial and cultural features are also observed at smaller, independent festivals and indeed whether other issues arise. Despite these limitations, our findings add to the mounting evidence that reveals sexual violence and misogyny are commonplace experiences for women in leisure spaces and we hope it will act as a call to festival organizers and the music industry more widely to take this issue seriously and prioritize prevention and support for survivors.
